# The first-line antihypertensive nitrendipine potentiated the therapeutic effect of oxaliplatin by downregulating CACNA1D in colorectal cancer

**DOI:** 10.1515/med-2024-1138

**Published:** 2025-02-12

**Authors:** Chengzhe Lai, Jinghu Liu, Jingna Zhou, Haokun Zhou

**Affiliations:** Department of Cardiology, The Fourth Affiliated Hospital of Guangzhou Medical University, Zengcheng, Guangzhou, China; Department of Medicine, Taizhou University, Zhejiang, China

**Keywords:** antihypertensive, nitrendipine, oxaliplatin, colorectal cancer, CACNA1D

## Abstract

**Background:**

Oxaliplatin (OXA) is among the most common chemotherapy drugs and is the base component of the FOLFOX regimen (OXA + leucovorin + 5-fluorouracil) and CapeOX regimen (OXA + capecitabine). Resistance to and failure of these two OXA-based regimens often results in poor outcomes in patients with colorectal cancer (CRC). Nitrendipine (NTD) is a first-line antihypertensive drug commonly used in hypertension and coronary heart disease with confirmed low toxicity and side effects. However, the potential benefits of NTD for CRC progression and therapy remain unclear.

**Methods:**

Cell counting kit-8 (CCK-8) detection, colony formation assay, wound-healing assay, Transwell assay, SynergyFinder webtool, and subcutaneous tumor models were used to assess the effect of NTD with OXA on CRC inhibition *in vitro* and *in vivo*. Bioinformatics tools including Human Protein Atlas (HPA), quantitative real-time polymerase chain reaction, western blotting analyses, lentivirus transfection, and rescue experiment were used to investigate the mechanism(s) of the related action.

**Results:**

Utilizing murine and human CRC cell lines, the *in vitro* and *in vivo* experiment demonstrated that NTD inhibited cell proliferation, migration, and invasion, and the synergy scores calculated by SynergyFinder indicated that NTD exhibited synergistic activity with the chemotherapeutic drug OXA. The CCK-8 detection, animal model, and rescue experiment results demonstrated that NTD suppressed CRC progression and potentiated OXA therapeutic effect by downregulating calcium voltage-gated channel subunit alpha1 D (CACNA1D).

**Conclusions:**

This study presents novel data on first-line antihypertensive NTD, exerting inhibitory effects on cell proliferation and migration in CRC and revealing synergistic activity with OXA by downregulating CACNA1D. NTD may be a candidate as a promising chemosensitizer as an OXA new combination to improve the efficacy and safety of CRC therapy.

## 1 Introduction

Colorectal cancer (CRC) remains a major public health problem worldwide and is the second most frequent malignancy, with a high case–fatality ratio [1]. Currently, surgery, chemotherapy, antiangiogenic therapy, and radiotherapy have greatly benefited patients with CRC by prolonging survival time, improving quality of life, and achieving a complete cure [2]. Many early-stage patients with CRC could receive considerable therapeutic benefits from surgery, such as total colorectal resection [3]. Nevertheless, due to the lack of easily distinguishable symptoms, precise diagnosis of early-stage CRC remains difficult, and most patients often have a confirmed diagnosis of advanced-stage CRC and need to receive combined therapeutic regimens, including surgery and chemotherapy [4,5]. The present situation may indicate that metastatic or advanced patients demonstrate a very poor 5-year survival rate of approximately 10% due to chemotherapy resistance and failure [6]. Therefore, there is an urgent need to identify and develop effective therapeutic strategies to reduce recurrence and improve CRC prognosis.

Oxaliplatin (OXA) is among the most common chemotherapy drugs used in advanced patients with CRC, which is the basic agent of the FOLFOX regimen (OXA + leucovorin + 5-fluorouracil) and CapeOx regimen (OXA + capecitabine). It markedly impedes CRC progression and brings considerable therapeutic benefits to patients with CRC [7,8,9]. However, long-term repeated use of OXA combination regimens often results in chemotherapy resistance and failure [10]. Moreover, the significant toxicities of the current chemotherapy regimens (CapeOx or FOLFOX), including gastrointestinal, hematological, and neurological toxicities, could further block the continuity of the therapy regimen [11,12]. Consequently, there is a need to identify and develop novel chemosensitizers as new OXA combinations to enhance overall clinical efficacy.

Nitrendipine (NTD) is a first-line antihypertensive agent of dihydropyridine calcium channel blockers (CCBs). It has been widely used in clinical practice for many years for its high efficiency and low toxicity [13]. Recently, many researchers have focused on exploring and identifying the potential anticancer activities of CCBs, such as amlodipine and nifedipine [14,15]. For example, Wu et al. [15] reported that another CCB, nifedipine, prevents immune escape and enhances immune checkpoint blockade in CRC. However, the role and clinical significance of the more commonly used CCBs NTD in CRC progression and chemotherapy remains largely unknown.

In this study, we aimed to explore the role of NTD in CRC progression in combination with OXA and to elucidate the specific molecular mechanisms that may be involved. Our results indicated that by downregulating the expression of calcium voltage-gated channel subunit alpha1 D (CACNA1D), NTD significantly inhibited the proliferation and migration of CRC cells and enhanced OXA efficacy *in vitro* and *in vivo*.

## 2 Materials and methods

### 2.1 Chemicals and reagents

The NTD was bought from Yuanye Biotechnology Co., Ltd (Cat # B27254, Shanghai, China). Oxaliplatin was obtained from Macklin Biotechnology Co., Ltd (Cat # O815347, Shanghai, China). NTD and OXA were dissolved in dimethyl sulfoxide and stored at –20°C.

### 2.2 Cell culture

The murine CRC cell line MC38 was procured from Bohui Biological Technology Co., Ltd (Cat # BH-C459, Guangzhou, China), and human CRC cell lines RKO, LOVO, SW-620, and HT29 were acquired from Bohui Biological Technology Co., Ltd (Cat # BH-C459, Cat # BH-C113, Cat # BH-C226, Cat # BH-C132, and Cat # BH-C062, Guangzhou, China) and were initially obtained from the American Type Culture Collection (Cat # CRL-2577, Cat # CCL-229, Cat # CCL-227, and Cat # HTB-38, Maryland, USA). FHC was procured from Meisen Biological Technology Co., Ltd (Cat # CTCC-001-0208, Guangzhou, China). MC38 cells were cultured in Dulbecco’s modified Eagle’s medium (DMEM, Cat # C11995500BT, Gibco, USA). In contrast, RKO and HT29 cells were cultured in Roswell Park Memorial Institute-1640 (RPMI-1640, Cat # C11875500BT, Gibco, USA) medium supplemented with 10% fetal bovine serum (FBS, Cat # 10091, Gibco, USA) and 1% penicillin–streptomycin (Cat # P1400, Solarbio, China). The culture medium for FHC was DMEM with 20% FBS. All cell lines were subjected to mycoplasma testing. Cells were cultured in an incubator with an atmosphere of 5% CO_2_ at 37°C.

### 2.3 Cell counting kit-8 (CCK-8) detection

MC38, RKO, and HT29 cells were seeded in 96-well plates at a density of 1,000 cells/well. OXA and NTD were diluted to gradient concentrations in the corresponding complete media. The previous medium was replaced with the prepared solution, and the cells were cultured in an incubator with an atmosphere of 5% CO_2_ at 37°C for 48 h. Finally, CCK-8 (Cat # CK04, Dojindo, Japan) was employed to examine the cell viability following the manufacturer’s protocol. After each well was replaced with the solution (CCK-8: culture medium = 1:9) and incubated for 4 h, a microplate reader was used to detect the optical density (OD) value of each well at 450 nm. GraphPad Prism software (version 9.0; GraphPad, La Jolla, CA, USA) was employed to calculate 50% inhibitory concentration (IC_50_) values.

### 2.4 Synergy determination with SynergyFinder

MC38, RKO, and HT29 cells were seeded into 96-well plates at 1,000 cells/well density and prepared for treatment as described below. Either single treatment alone (NTD, OXA) or combinations (NTD and OXA) were analyzed using the indicated amounts from the CCK-8 detection above. The IC_50_ value of each drug was calculated as the concentration gradient (NTD concentration: 25, 50, 100, 250, and 500 μM; OXA concentration: 3.125, 6.25, 12.5, 25, and 50 μM). After 48 h of treatment, the CCK-8 kit was utilized to examine cell viability on a microplate reader (Cat # 1681135, Bio-Rad Laboratories Inc., USA), as described above. The OD value of each well at 450 nm was determined using EXCEL software and was ready for the next analysis. By utilizing the response surface model and zero interaction potency (ZIP) calculation method, the online software SynergyFinder (https://synergy-finder.fimm.fi) was employed to calculate drug synergy scoring with the “inhibition index” (the inhibition index = 100 – cell viability). When the ZIP synergy scores were greater than 0, the synergy determination was synergism (red regions), and scores greater than 10 were strongly synergistic. Heat maps of drug synergistic responses were also plotted to reveal the therapeutic significance of the combination [16].

### 2.5 Colony formation assay

MC38 and RKO cells were seeded in six-well plates at 600 cells/well density and cultured in an incubator with an atmosphere of 5% CO_2_ at 37°C for 24 h. The culture medium was replaced with a complete medium containing NTD or OXA. Finally, the colonies were fixed with 4% paraformaldehyde and stained with the Giemsa dye for 30 min. ImageJ software was used to count the number of stained colonies.

### 2.6 Wound-healing assay

RKO cells were seeded in six-well plates at a density of 2 × 10^6^ cells/well. The cells were grown into a monolayer. A small pipette tip was used to create a scratch wound in each well. At 0, 12, 24, and 36 h after treatment, photos of three randomly selected microscopic fields (×200) were taken to evaluate the migration status of the cells. The wound healing area was calculated using ImageJ software, and the ratio of cell migration was calculated.

### 2.7 Animal experiments

C57BL/6J female mice (5–6 weeks old, 19–20 g) were procured from Guang Dong Model Biotechnology Co., Ltd (China) and bred under specific pathogen-free conditions. To establish the CRC model, MC38 cells were subcutaneously injected into the right flank of mice at a density of 5 × 10^5^ cells per mouse. When the tumor volume approached 50–100 mm^3^, the mice were randomly assigned to four groups (control, NTD treatment alone, OXA treatment alone, and combination treatment groups). The mice received OXA (3 mg/kg/2 days, intraperitoneally [i.p.]) and NTD (25 mg/kg/2 days, i.p.). Two to three weeks after cell injection, the tumors were harvested when the tumor mass approached approximately 1,000 mm^3^ (less than 1,500 mm^3^) or when obvious ulceration or cachexia occurred. The procedure refers to the material and method used in a recent study [15,16].

### 2.8 Quantitative real-time polymerase chain reaction (qPCR) assay

Control and treated cells or tumor tissues were collected at a low temperature. The cells were lysed using TRIzol reagent (Cat # 15596026CN, Invitrogen, USA), and total RNA was extracted according to the manufacturer’s protocol. Prime-Script RT Reagent Kit (Cat # RR037A, Promega, Madison, WI, USA) was employed to reverse-transcribed the mRNA into cDNA. SYBR Green PCR Mix (Cat # K1070, Apexbio, USA) was used for qPCR using a Roche LightCycler 480 Real-Time PCR System (Cat # LightCycler 480 II, Roche Life Science, USA). GAPDH was used as an internal control. The primer sequences for GAPDH amplification were as follows: forward primer: GGAGCGAGATCCC-TCCAAAAT; reverse primer: GGCTGTTGTCATAC TTCTCATGG. The primer sequences for CACNA1D amplification were as follows: forward primer: CGCGAACGAGGCAAACTATG; reverse primer: TTGGAGCTATTCG-GCTGAGAA. The relative expression levels of target genes were normalized using the 2^–ΔΔCt^ method. The experiments were conducted three times in the same procedure.

### 2.9 Western blotting analysis

Total cell proteins were collected, lysed, separated by sodium dodecyl sulfate-polyacrylamide gel electrophoresis, and electro-transferred onto a polyvinylidene difluoride membrane. Then, the membranes were blocked with 5% skimmed milk and incubated using primary antibodies against GAPDH (1:1,000 dilution, Cat # RM2002, Beijing Ray Antibody Biotech), anti-CACNA1D (1:500 dilution, Cat # ab85491, Abcam, USA), followed by incubation with the appropriate secondary antibodies. An enhanced chemiluminescence (Cat # 750L, Pierce, Rockford, IL, USA) was used to detect signals.

### 2.10 RNA interference and lentivirus transfection

The human siRNA sequence targeting CACNA1D (siCACNA1D) was GCGTGCTATATCGTGTGATTT (Cat # siBDMV002-10, Ribobio, China). RKO cells were successfully transfected with siRNA using Lipofectamine 2000 (Cat # 11668030, Thermo Fisher, USA). The murine shRNA sequences targeting CACNA1D (shCACNA1D) were CCTGCATTAGTATAGTGGAAT (Cat # S24042008, Cyagen, Suchow, China). PCDNA3.1-mouse-CACNA1D was obtained from Vigene (Cat#WZ010001, Shan Dong, China). First, package tool cells HEK293 were transfected with plasmids using Lipofectamine 2000 for 24 h. The medium was then replaced with DMEM supplemented with 10% FBS. After 48 h, the targeted recombinant lentiviruses were collected and transfected into the CRC cells. The knockdown efficiency was examined by qPCR.

### 2.11 Bioinformatics analysis

The mRNA expression of CACNA1D generated by TCGA was obtained from the Gene Expression Profiling Interactive Analysis 2 (GEPIA2) database (http://gepia2.cancer-pku.cn/#index), and the protein expression of CACNA1D in patients with CRC was obtained from the Human Protein Atlas (HPA) database.

### 2.12 Statistical analysis

All statistical analyses were performed using SynergyFinder and GraphPad Prism (version 9.0) software. Numerical data are presented as mean ± standard deviation. The statistical significance of the differences was analyzed using Student’s and unpaired *t*-tests according to the test of homogeneity of variances. Survival rates were evaluated using the Kaplan–Meier method and tested using the log-rank test. Error bars represent the standard error of the mean; n.s. no significance; *p < 0.05; **p < 0.01; ***p < 0.001; *p* < 0.0001. *p* < 0.05 was considered statistically significant.


**Ethical statement:** The animal study was in accordance with the National Institutes of Health Guide for the Care and Use of Laboratory Animals and reviewed and approved by Taizhou University.

## 3 Results

### 3.1 NTD inhibited the cell proliferation and migration viability of CRC cells

**Figure 1:**
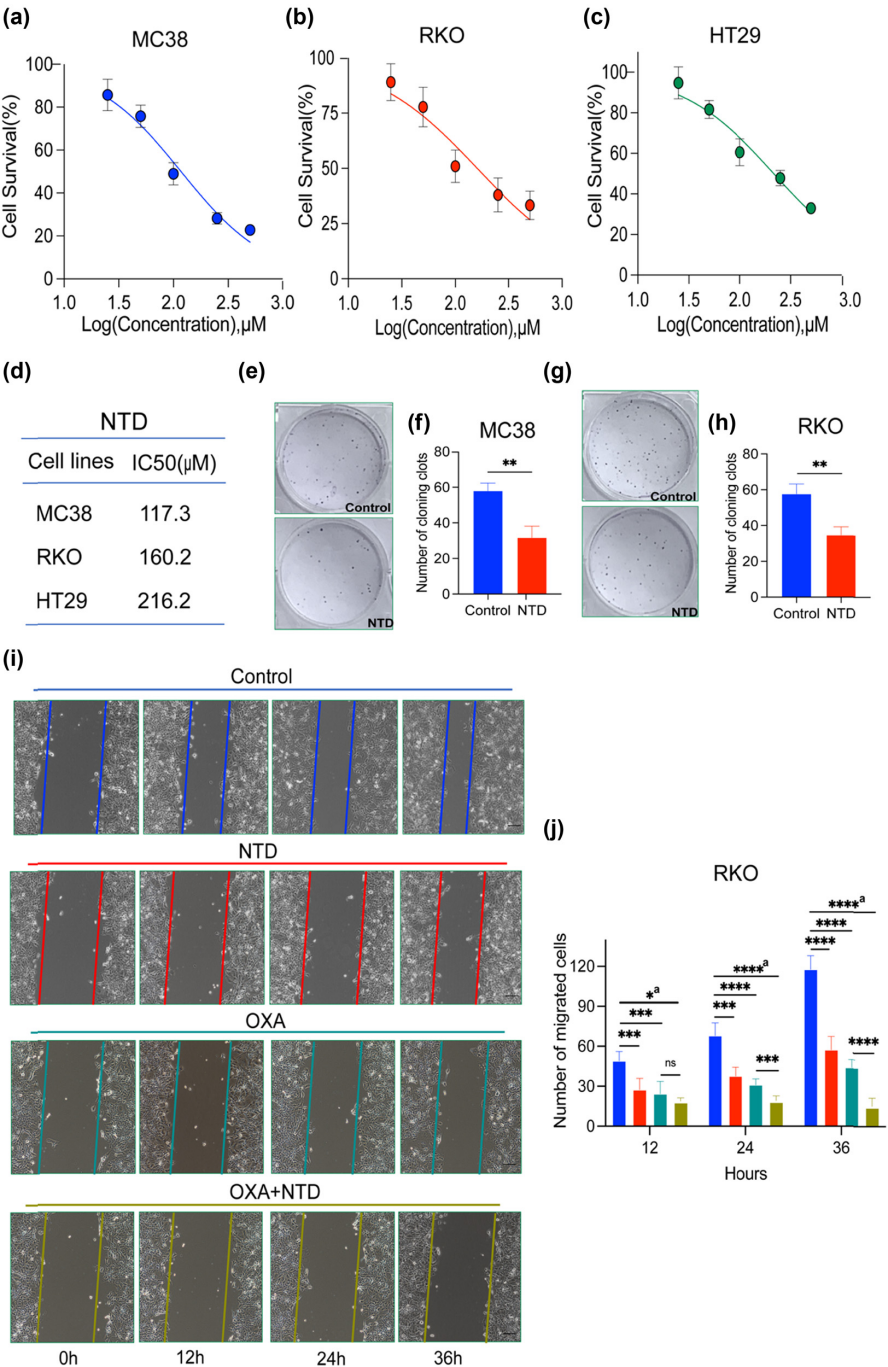
NTD inhibited the cell proliferation and migration viability of CRC cells. (a)–(c) Dose–response examination of NTD in MC38 (a), RKO (b), and HT29 cells (c). (d) IC_50_ values of NTD for MC38, RKO, or HT29. (e) and (f) NTD (100 μM) suppressed the ability of colony formation of MC38 cells. (g) and (h) NTD (100 μM) suppressed the ability of colony formation of RKO cells. (i) and (j) NTD (100 μM) and OXA (6.25 μM) inhibited the migration ability of RKO cells. a, analysis of variance performed between multiple groups.

The CCK-8 assay was used to assess the biological effect of NTD in a concentration gradient on the murine and human CRC cell lines MC38, RKO, and HT29. As demonstrated by the dose–response curves, NTD exerted an inhibitory effect on each cell line in a concentration-dependent manner (Figure 1(a)–(c)). The IC_50_ value was also calculated and was displayed in Figure 1(d). We also found that the non-cytotoxic concentration of NTD was not more than 100 μM (Figure S1(a)). Next, we performed a colony formation assay to confirm the antiproliferative effect of NTD on CRC cells. The result indicated that NTD highly inhibited colony formation in the murine CRC cell lines MC38 and CT26 (Figure 1(e) and (f) and Figure S1(d) and (e)) and the human CRC cell line RKO and HT29 (Figure 1(g) and (h) and Figure S1(b) and (c)). Furthermore, NTD not only illustrated an inhibiting effect on RKO invasion but also exerted strong synergistic preventive effect on OXA inhibition on RKO migration and invasion (Figure 1(i) and (j), Figure S1(f)–(i)). In conclusion, NTD inhibits the proliferation and migration of CRC cells *in vitro.*

### 3.2 The combination of NTD plus OXA synergistically enhanced CRC inhibition *in vitro* and *in vivo*

**Figure 2:**
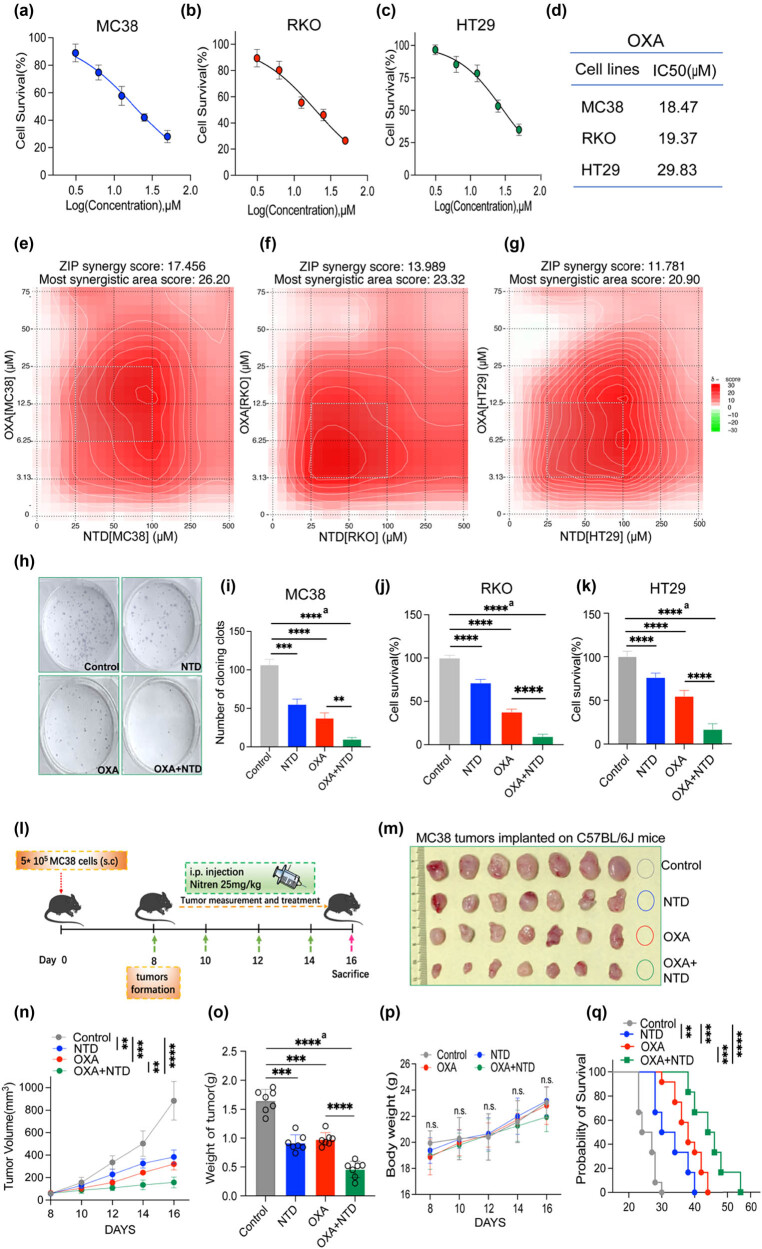
NTD synergistically enhances the cytotoxicity of OXA against CRC cells. MC38 (a), RKO (b), and HT29 cells (c). (d) IC_50_ values of OXA for MC38, RKO, or HT29. (e)–(g) Heatmaps of drug combination responses. NTD and OXA synergistically inhibited MC38 (e), RKO (f), or HT29 (g) cell growth *in vitro*. MC38, RKO, or HT29 cells were treated with NTD and OXA at the indicated concentrations for 48 h, and CCK-8 was employed to detect cell viability. SynergyFinder software was used to calculate the ZIP Synergy scores. Scores > 0: synergism, and scores > 10: strongly synergistic. The gradation of the red regions revealed synergism intensity. The white rectangle displayed the concentrations encompassing the region of highest synergy, and the *X*- and *Y*-axes corresponding to the sides of the white rectangle demonstrate the concentrations at which the drug combination exerts the maximum inhibitory impact on cell proliferation. (h) and (i) The combination of OXA (12.5 μM) plus NTD (100 μM) enhanced the colony formation of MC38 cells. (j) CCK-8 detection of cell viability of RKO cells after being treated with OXA (12.5 μM) plus NTD (100 μM) for 48 h. (k) CCK-8 detection of cell viability of HT29 cells after being treated with OXA (12.5 μM) plus NTD (100 μM) for 48 h. (l) The scheme of NTD and OXA treatment on MC38 subcutaneous tumor model (control group [*n* = 7], NTD group [*n* = 7], OXA group [*n* = 7], OXA + NTD group [*n* = 7]). (m) Photo of MC38 subcutaneous tumor. (n) Growth of curve of tumor volume. (o) Weight of tumor. (p) Weight of mice. (q) The survival rate of MC38-tumor-bearing mice received combination therapy of NTD and OXA. a, analysis of variance performed between multiple groups.

The CCK-8 assay was also employed to assess the toxicity of OXA in MC38, RKO, and HT29 cells, and OXA displayed concentration-dependent antiproliferative effects in CRC cells (Figure 2(a)–(c)). The IC_50_ values were also calculated and are displayed in Figure 2(d). Using the online SynergyFinder software, the drug ZIP synergy scores of MC38, RKO, and HT29 were calculated according to other concentration gradients and the corresponding inhibition index (Figure 2(e)–(g)). The results demonstrated that the average (and maximum) proportions of the antiproliferative effect attributable to drug interaction were 17.456 (26.20) in MC38 cells, 13.989 (23.32) in RKO cells, and 11.781(20.92) in HT29 cells. Indeed, the combined treatment of NTD and OXA displayed significant synergistic effects in antiproliferative activity (ZIP synergy scores >0). As depicted in Figure 2(e)–(g), the white rectangle indicates the region of the maximum synergistic area. The results indicated that the concentration of NTD encompassing the region of highest synergy was between 25 and 100 μM. The lowest effective and best-combined concentration of NTD was determined as 25 μM. The results also indicated that concentrations of OXA, 6.25–25 μM for MC38 cells and 3.13–12.5 μM for RKO cells, may be the best concentrations to display synergistic antiproliferative effects encompassing the region of highest synergy.

Next, we performed a colony formation assay to further confirm the best-combined concentration of NTD and OXA for inhibiting cell proliferation *in vitro*. The results demonstrated that 100 μM NTD and 12.5 μM OXA highly synergistically inhibited colony formation in the murine CRC cell line MC38 (Figure 2(h) and (i)). A similar outcome was observed using CCK-8 detection in the human CRC cell line RKO by adding NTD at 100 μM and OXA at 12.5 μM (Figure 2(j)) and HT29 by adding NTD at 100 μM and OXA at 12.5 μM (Figure 2(k)). Using a murine subcutaneous CRC model (Figure 2(l)), we found that the NTD and OXA groups significantly suppressed tumor growth *in vivo* compared to the control group. Compared to the OXA group, the NTD + OXA group revealed enhanced tumor growth inhibition (Figure 2(m)–(o)). Furthermore, NTD treatment alone significantly inhibited other tumors, such as melanoma (Figure S1(j)–(l)). NTD-enhanced immune checkpoint inhibitors programmed death-1 antibody therapeutic effect on CT26 tumors *in vivo* (Figure S1(m)–(o)). The weight of mice in each group revealed a non-significant change (Figure 2(p)). Notably, NTD treatment alone and in combination with OXA significantly prolonged survival time in tumor-bearing mice (Figure 2(q)).

Collectively, the combination of NTD plus OXA enhanced cell proliferation and migration inhibition *in vitro* and *in vivo.*

### 3.3 NTD inhibited CRC progression and enhanced OXA therapeutic effect *in vitro* by downregulating CACNA1D

**Figure 3:**
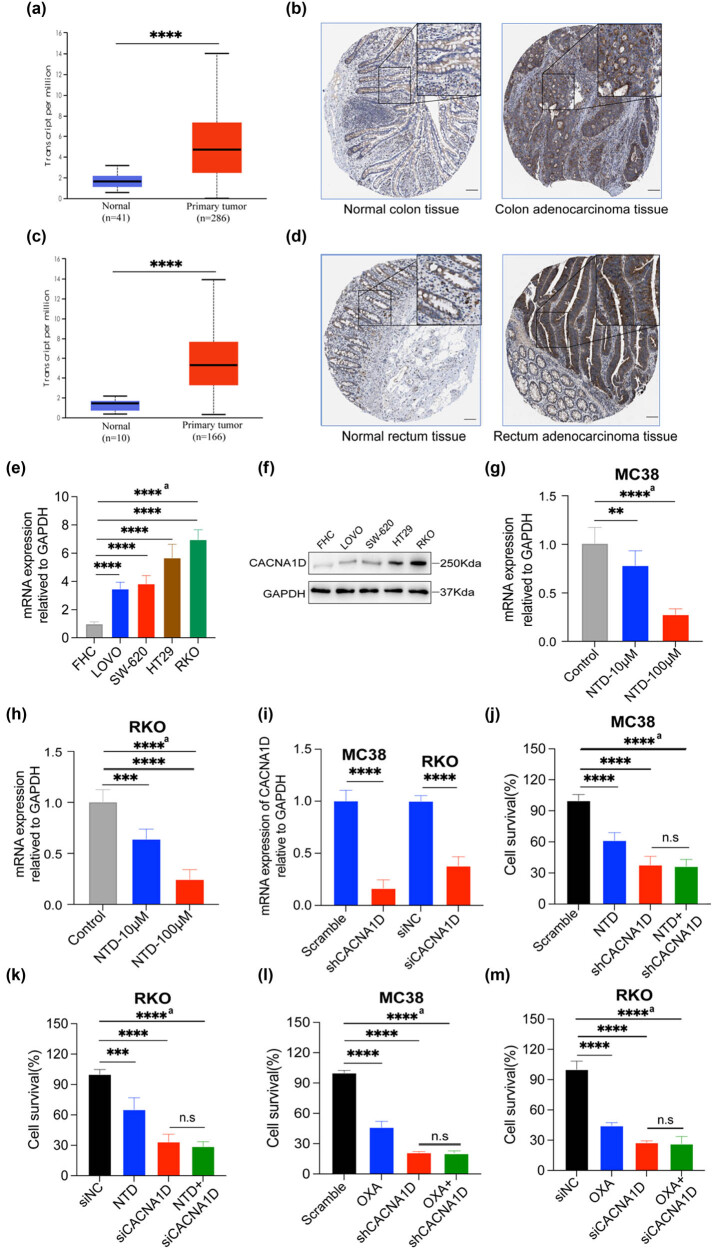
NTD inhibited CRC progression and enhanced OXA therapeutic effect *in vitro* by downregulating CACNA1D. (a) Data from the GEO were utilized to analyze CACNA1D expression in colon tumor tissues. (b) IHC detection data of CACNA1D expression in colon tumor tissues downloaded from the HPA database (www.proteinatlas.org), scale bar, 100 μm. (c) Data from the GEO were utilized to analyze the CACNA1D expression in rectum tumor tissues. (d) IHC detection data of CACNA1D expression in rectum tumor tissues downloaded from the HPA database (www.proteinatlas.org), scale bar, 100 μm. (e) qPCR detection of CACNA1D expression of FHC and CRC cell lines. (f) WB detection of CACNA1D expression of FHC and CRC cell lines. (g) qPCR detection of CACNA1D expression in MC38 after NTD treatment. (h) qPCR detection of CACNA1D expression in RKO after NTD treatment. (i) The efficiency of CACNA1D knockdown in MC38 and RKO was examined by qPCR assay. (j) Cell viability of MC38 after CACNA1D knockdown and with the addition of NTD (100 μM) for 48 h. (k) Cell viability of RKO after knocking down CACNA1D and being treated by NTD (100 μM) for 48 h. (l) Cell viability of MC38 after CACNA1D knockdown and upon with the addition of OXA (20 μM) for 48 h. (m) Cell viability of RKO after CACNA1D knockdown and treated by OXA (20 μM) for 48 h. a, analysis of variance performed between multiple groups.

It has recently been documented that another antihypertensive agent and a CCB, nifedipine, could suppress prostate cancer progression by modulating CACNA1D expression [17]. Therefore, it is difficult to avoid the hypothesis that CACNA1D participates in the mechanism by which NTD prevents CRC progression. Using HPA and GEPIA2 web tools, we found that CACNA1D expression was higher in colon tumor tissues than in normal tissues (Figure 3(a) and (b)). Moreover, CACNA1D expression was higher in rectal tumor tissues than in normal tissues (Figure 3(c) and (d)). The qPCR and WB results confirmed that compared to normal colonic epithelial cells FHC, CRC cell lines such as RKO, HT29, SW-620, and LOVO exhibited higher expression of CACNA1D (Figure 3(e) and (f)). Furthermore, the concentration gradient of NTD decreased CACNA1D expression in the murine CRC cell line MC38 and the human CRC cell line RKO (Figure 3(g) and (h)). After successfully knocking down CACNA1D in MC38 and RKO cells (Figure 3(i), Figure S2(a)), we performed a CCK-8 assay to assess cell viability upon the addition of NTD and OXA. The results suggested that the knockdown of CACNA1D and NTD significantly impaired the MC38 cell growth when compared to the control group. However, when compared with the shCACNA1D group, the cell viability of the NTD + shCACNA1D group was non-significantly different (Figure 3(j)). A similar outcome was observed in the human CRC cell line RKO (Figure 3(k)). When OXA was added, the results indicated that OXA significantly suppressed cell proliferation when compared to the control group. Nevertheless, compared with the shCACNA1D group, the OXA + shCACNA1D group revealed a non-significant inhibitory effect (Figure 3(l)). In other words, NTD did not suppress cell growth after CACNA1D knockdown. A similar outcome was observed in the human CRC cell line RKO (Figure 3(m)). Furthermore, the results of rescue experiments indicated that the application of CACNA1D overexpression could rescue the inhibitory effect of NTD exerting on the cells *in vitro* and *in vivo* (Figure S2(b)–(f)). Collectively, these results indicated that NTD may inhibit CRC progression and enhance OXA therapeutic effects *in vitro* by downregulating CACNA1D.

### 3.4 NTD potentiated OXA inhibition on CRC *in vivo* by downregulating CACNA1D

**Figure 4:**
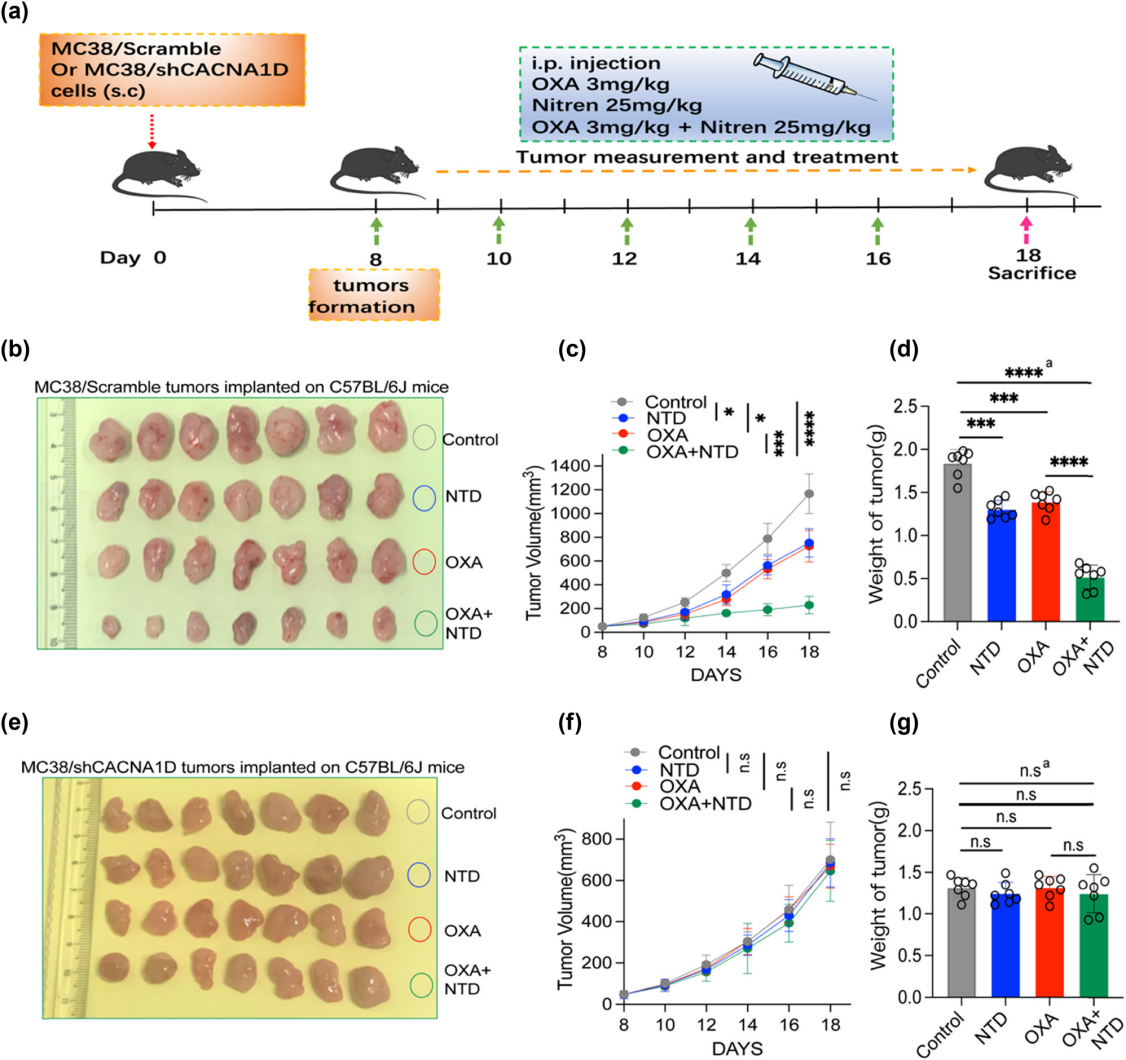
NTD potentiates OXA inhibition on CRC *in vivo* by downregulating CACNA1D. (a) The scheme of scramble and shCACNA1D MC38 subcutaneous tumors after receiving combination therapy of NTD and OXA. (b) Photo of MC38/scramble group subcutaneous tumor(control group [*n* = 7], NTD group [*n* = 7], OXA group [*n* = 7], OXA + NTD group [*n* = 7]). (c) Growth curve of MC38/scramble group tumor. (d) Weight of MC38/scramble group tumor. (e) Photo of MC38/shCACNA1D group subcutaneous tumor (shCACNA1D group (*n* = 7), NTD + shCACNA1D group (*n* = 7), OXA + shCACNA1D group (*n* = 7), and OXA + NTD + shCACNA1D group (*n* = 7)). (f) Growth curve of MC38/shCACNA1D group tumor. (g) Weight of MC38/shCACNA1D group tumor. a, analysis of variance performed between multiple groups.

To further validate the role of CACNA1D in inhibiting CRC progression and enhancing the therapeutic effects of OXA, we used a syngeneic murine CRC model in this section (Figure 4a). The results demonstrated that in the model established using MC38/scramble cells, compared to the control group, NTD and OXA alone significantly suppressed the growth of MC38 tumors, and the combination treatment displayed the strongest inhibition (Figure 4(b)–(d)). However, when CACNA1D was successfully knocked down, the antitumor effect of NTD + OXA was impaired *in vitro* (Figure S2(g)). In the model established using MC38/shCACNA1D cells, the tumor growth rate and weight of NTD or OXA groups were comparable to those of the control group. Besides, the tumor size and weight in the NTD + OXA group did not differ from that of the OXA group or the NTD group (Figure 4(e)–(g)). Therefore, OXA administration revealed a non-significant increase in antitumor effect in the MC38/shCACNA1D model. The results of qPCR detection of tumor samples revealed that when compared with the control group, the mRNA expression of CACNA1D in the NTD group and OXA group decreased, and the combination group remained the lowest. When CACNA1D was successfully knocked down, the mRNA expression of CACNA1D was significantly decreased, and the mRNA expression of CACNA1D in the NTD group or the OXA group or the combination group was comparable to those of the control group (Figure S2(h)). In summary, NTD potentiated OXA inhibition and enhanced the OXA therapeutic effect in CRC *in vivo* by downregulating CACNA1D expression.

## 4 Discussion

This study demonstrates that the first-line antihypertensive NTD arrested the progression of CRC by suppressing cell proliferation and migration and revealed synergistic activity with the commonly used chemotherapy drug OXA by downregulating CACNA1D. This is the first study to explore the activity and mechanism of NTD in CRC *in vivo* and *in vitro*. In conclusion, these findings indicate that combination therapy of NTD plus OXA may be a promising clinical treatment for CRC.

Cancer is a leading cause of mortality worldwide. Together, hypertension is recognized as among the most common comorbidities in patients with cancer, such as CRC, owing to overlapping epidemiologic features, such as most old patients. Furthermore, several reports have indicated that hypertension is closely associated with the incidence and prognosis of common malignancies, including breast, kidney, colorectal, oral, laryngeal, and esophageal cancers [18,19,20,21]. Patients with cancer and hypertension typically have poorer clinical outcomes than normotensive ones. Therefore, it is important to identify the role of common first-line antihypertensive NTD in cancer treatment. The present study may assist in solving these problems: (1) it helps oncologists choose a reasonable antihypertensive NTD to control blood pressure when prescribing OXA-based chemotherapy regimens like (FOLFOX or CapeOx). (2) The present research may provide novel insights into the development of other commonly prescribed, non-antitumor agents for cancer therapy.

NTD is a very commonly used antihypertensive that belongs to a CCB demonstrated to prevent the movement of calcium through the “slow channel” of cardiac and vascular smooth muscle, resulting in peripheral vasodilation with a consequent reduction in elevated blood pressure [13,22]. Recently, many researchers have focused on the antitumor action and related mechanisms of CCBs. CCBs, including verapamil, nifedipine, and felodipine, have been demonstrated to exert antitumorigenic effects in breast, gastric, prostate, ovarian, and lung cancers [23,24,25,26]. However, previous reports on CCBs in cancer chemotherapy are limited, predominantly focusing on the synergistic effect of verapamil with chemotherapeutic agents such as paclitaxel, adriamycin, and doxorubicin against various cancers [27,28,29]. However, there is a paucity of studies on other commonly used antihypertensive NTD. Compared to verapamil, NTD is more commonly used and is generally suitable for patients with hypertension and coronary heart disease because it has a protective effect on the ischemic myocardium [30]. Nevertheless, laboratory research on NTD for cancer prevention and therapy is scarce. In this study, we found that NTD suppressed CRC progression by inhibiting cell proliferation and migration. Surprisingly, NTD revealed synergistic activity with the chemotherapeutic drug OXA *in vitro* and *in vivo*. Our research suggested that NTD may be a novel chemosensitizer as a new OXA combination to improve the efficacy and safety of CRC therapy. However, NTD has potential side effects such as hypotension, headache, and facial flushing [13]. The adverse events of OXA most often cited were hematologic toxicity, gastrointestinal tract toxicity, and neuropathy unlike that observed with other platinum derivatives [31]. Therefore, the potential side effects should be brought to clinicians’ attention when using NTD in combination with OXA for the treatment of CRC with high CACNA1D expression.

Aberrant Ca^2+^ signaling has been reported to participate in malignant biological properties such as proliferation, invasion, and migration [32]. In this pathway, ion channels, such as CaV channels, which comprise pore-forming α1 subunits and a series of accessory/regulatory β and α2δ subunits, play vital roles in disease progression or treatment resistance [33,34]. Among the CaVs, CaV1.3, encoded by CACNA1D, was recently reported to be upregulated in cancer tissues compared to related normal tissues [35]. Laboratory studies have reported that CaV1.3 overexpression significantly promotes breast, prostate, and gastric cancer progression [36,37,38]. For example, McKerr et al. reported that CACNA1D overexpression potentiated the malignancy of prostate cancer and that CCBs nifedipine can reverse this phenomenon [17]. However, the role of CACNA1D in CRC progression and therapy remains unclear. In this study, using bioinformatics tools, we found that CACNA1D was upregulated in CRC tissues than normal tissues, and the CACNA1D expression was negatively correlated with prognosis. Furthermore, the CCBs NTD introduction decreased the CACNA1D expression dose-dependently. After the successful knockdown of CACNA1D, compared to the shCACNA1D group, cell proliferation in the shCACNA1D + NTD and shCACNA1D + NTD + OXA groups displayed no significant changes *in vitro* and *in vivo*; These findings suggest that CACNA1D may play a key role in NTD inhibition and in combination with OXA.

In conclusion, this study presents novel data on first-line antihypertensive NTD exerting inhibitory effects on cell proliferation and migration of CRC and reveals synergistic activity with OXA by downregulating CACNA1D. This study offers a theoretical basis for combining OXA with NTD for CRC treatment. However, some limitations cannot be neglected, as more direct evidence is needed to verify that NTD suppresses CRC progression through CACNA1D. Detailed mechanistic studies will be conducted in the future.

## Supplementary Material

Supplementary Figure
