# Supplementary material for: The first-line antihypertensive nitrendipine potentiated the therapeutic effect of oxaliplatin by downregulating CACNA1D in colorectal cancer
Source: Open Med (Wars). 2025 Feb 12;20(1):20241138. doi: 10.1515/med-2024-1138 (PMC11826243; doi:10.1515/med-2024-1138)
Supplement: Supplementary Figure [file med-2024-1138-sm.pdf]

Supplementary material

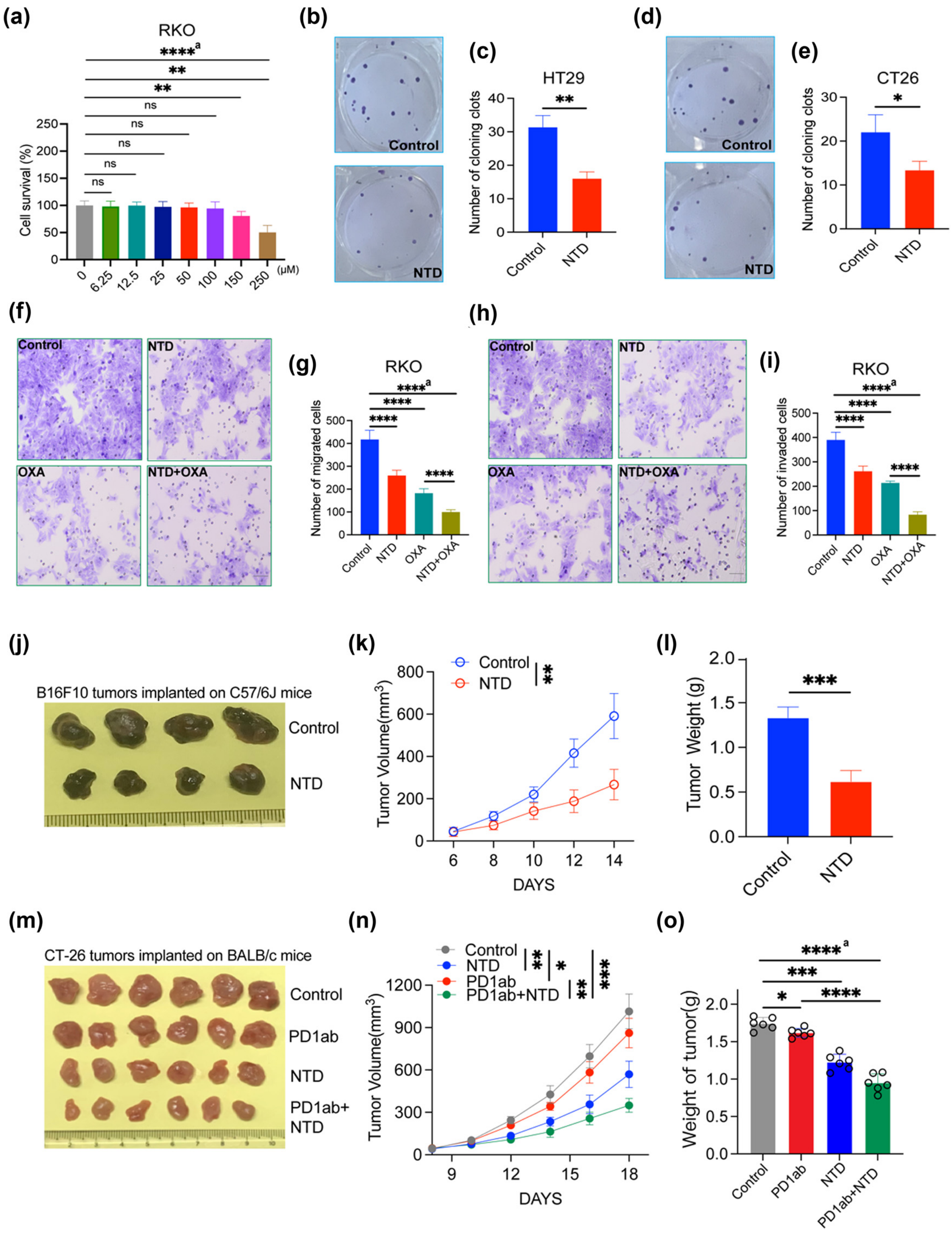

Figure S1: (a) Cell viability of RKO after NTD treatment with different concertation for 48 h; (b) and (c) NTD (100 μM) suppressed the ability of colony formation of HT29 cells; (d) and (e) NTD (100 μM) suppressed the ability of colony formation of CT26 cells; (f) and (g) Transwel assay detected the migration ability of RKO cells after NTD (100 μM) and OXA (6.25 μM) treatment; (h) and (i) Transwel assay detected the invasion ability of RKO cells after NTD (100 μM) and OXA (6.25 μM) treatment; (j) Photo of B16F10 subcutaneous tumor after NTD (25 mg/kg, i.p, every other day) treatment (Control group(n = 4), NTD group (n = 4)); (k) Growth of curve of B16F10 tumor volume; (l) Weight of B16F10 tumor; (m) Photo of CT-26 subcutaneous tumor after NTD (25 mg/kg, i.p, every other day) treatment and PD1ab (100 μg per mouse, i.p, every other day, a total of 500 μg per mouse) (Control group (n = 6), PD1ab group (n = 6), NTD group (n = 6), PD1ab + NTD group (n = 6)); (n) Growth of curve of CT26 tumor volume; (o) Weight of CT26 tumor; a, analysis of variance performed between multiple groups.

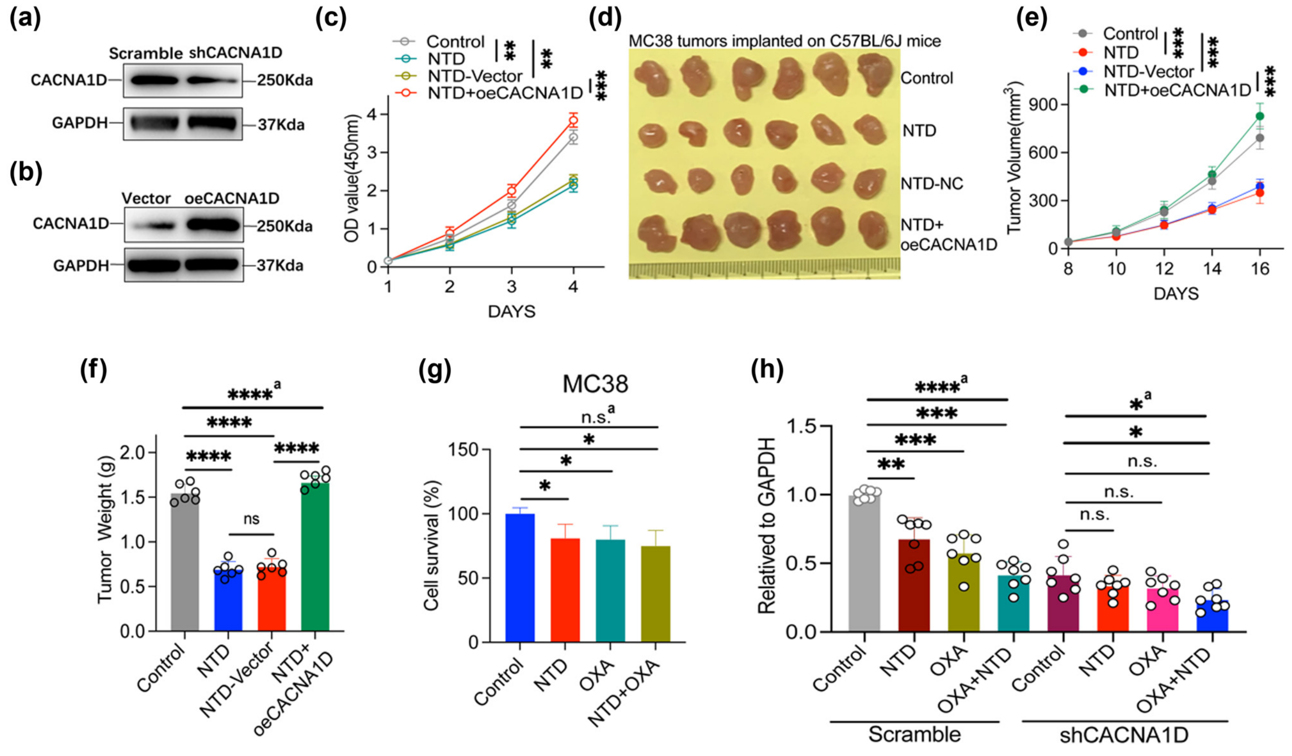

Figure S2: (a) The efficiency of CACNA1D knock down detected by WB assay. (b) The efficiency of CACNA1D overexpression detected by WB assay. (c) Cell viability of MC38-oeCACNA1D after NTD (100 μM) treatment. (d) Photo of MC38-oeCACNA1D subcutaneous tumor after NTD (25 mg/kg) treatment (Control group (n = 6), NTD group (n = 6), NTD + Vector group (n = 6), NTD + oeCACNA1D group (n = 6). (e) Growth of curve of MC38 tumor volume. (f) Weight of MC38 tumor. (g) qPCR detection of CACNA1D expression MC38-shCACNA1D tumor after NTD and OXA treatment. (h) Cell viability of MC38-shCACNA1D after being treated with OXA (12.5 μM) plus NTD (25 μM) for 48 h. [a]ANOVA analysis performed between multiple groups.
